# Autosomal *sdY* Pseudogenes Explain Discordances Between Phenotypic Sex and DNA Marker for Sex Identification in Atlantic Salmon

**DOI:** 10.3389/fgene.2020.544207

**Published:** 2020-10-14

**Authors:** Fernando Ayllon, Monica Favnebøe Solberg, François Besnier, Per Gunnar Fjelldal, Tom Johnny Hansen, Anna Wargelius, Rolf Brudvik Edvardsen, Kevin Alan Glover

**Affiliations:** ^1^Institute of Marine Research, Bergen, Norway; ^2^Department of Biological Sciences, University of Bergen, Bergen, Norway

**Keywords:** sex, evolution, autosome, pseudo-gene, SNP, Atlantic salmon, *sdY*, sex chromosome

## Abstract

Despite the key role that sex-determination plays in evolutionary processes, it is still poorly understood in many species. In salmonids, which are among the best studied fishes, the master sex-determining gene sexually dimorphic on the Y-chromosome (*sdY*) has been identified. However, *sdY* displays unexplained discordance to the phenotypic sex, with a variable frequency of phenotypic females being reported as genetic males. Multiple sex determining loci in Atlantic salmon have also been reported, possibly as a result of recent transposition events in this species. We hypothesized the existence of an autosomal copy of *sdY*, causing apparent discordance between phenotypic and genetic sex, that is transmitted in accordance with autosomal inheritance. To test this, we developed a qPCR methodology to detect the total number of *sdY* copies present in the genome. Based on the observed phenotype/genotype frequencies and linkage analysis among 2,025 offspring from 64 pedigree-controlled families of accurately phenotyped Atlantic salmon, we identified both males and females carrying one or two autosomal copies of *sdY* in addition to the Y-specific copy present in males. Patterns across families were highly consistent with autosomal inheritance. These autosomal *sdY* copies appear to have lost the ability to function as a sex determining gene and were only occasionally assigned to the actual sex chromosome in any of the affected families.

## Introduction

Most eukaryotic organisms reproduce sexually, yet the nature of the sexual system and the mechanism of sex determination often vary remarkably, even among closely related species (Ashman et al., 2014; Pennell et al., 2018). This is particularly true for teleosts where some species display genetic sex determination, some display environmental sex determination, while others a mixture of both (Heule et al., 2014). Furthermore, heterogametic systems for both male (XX females and XY males) and females (ZZ males and ZW females) are even found in closely related species of tilapias (Cnaani et al., 2008) or sticklebacks (Ross et al., 2009).

Atlantic salmon (*Salmo salar*) is an anadromous fish inhabiting temperate streams in the North Atlantic. It belongs to the family Salmonidae, which includes multiple species from 11 genera including salmon, trout, charr, freshwater whitefishes, ciscoes, and graylings. Globally, Atlantic salmon represents one of the most economically significant and iconic species, providing extensive angling recreation, large aquaculture production, and symbolizing healthy ecosystems in the rivers it inhabits. As a consequence, it is also one of the most exhaustively studied fish. The Atlantic salmon’s ancestor underwent a whole-genome duplication event approximately 88–103 million years ago (Macqueen and Johnston, 2014), and is now in the process of rediploidization. As a result of this process, the Atlantic salmon genome consists of many paralogous regions (Lien et al., 2016) which in principle can diversify (Kjaerner-Semb et al., 2016) and acquire new functions as has been observed in other species displaying duplicated genomes (Qian and Zhang, 2014). Interestingly, the presence of transposable elements found in the genome is among the highest found in vertebrates (Lien et al., 2016).

The master sex determining (MSD) gene in salmonids is *sexually dimorphic on the Y-chromsome* (*sdY*), and was first discovered in rainbow trout (*Oncorhynchus mykiss*) (Yano et al., 2012, 2013). The discovery of *sdY*, and the subsequent development of molecular assays for rapid genetic sex determination has opened novel possibilities. For example, assays have been used to determine genetic sex in adults that were not phenotyped but subsequently used for sex-specific studies such as investigation into the genetic basis of age at maturity (Ayllon et al., 2015; Barson et al., 2015; Kusche et al., 2017; Ayllon et al., 2019). However, several studies have reported a discordance between phenotypic and *sdY* sex within the Salmonidae family (Eisbrenner et al., 2014; Cavileer et al., 2015; Larson et al., 2016; Podlesnykh et al., 2017). Discordance between DNA markers for sex and phenotypic sex is not uncommon in fishes, and it is typical in a species displaying a combination of genetic and environmental sex determination (Hattori et al., 2019). However, environmental sex determination has not been reported in the family Salmonidae, and several alternative theories for this discordance have been put forward including phenotyping errors (Yano et al., 2013; Eysturskarð et al., 2017), sex reversal (Nagler et al., 2001; Williamson and May, 2002; Metcalf and Gemmell, 2006), loss of gene function (Podlesnykh et al., 2017), or dose effects (Brown et al., 2020) among others (Guyomard et al., 2014; Larson et al., 2016; King and Stevens, 2020). Nevertheless, the mechanisms underpinning this discordance are still unclear. Adding to the complexity of the situation is the fact that *sdY* has been mapped to different regions of the genome in the various salmonid species, but also within the same species, suggesting that it transposes to a new location either at the time of speciation (Phillips, 2013) or more recently within species (Kijas et al., 2018). Specifically within Atlantic salmon, *sdY* has been mapped to chromosomes Ssa02, Ssa03, Ssa06, and possibly Ssa21 (Eisbrenner et al., 2014; Lubieniecki et al., 2015; Kijas et al., 2018; Gabian et al., 2019) evidencing its transposition ability (Lubieniecki et al., 2015).

In this study, we have identified why the presence of *sdY* does not always correlate with maleness in Atlantic salmon. We first asked the question whether the observed discordance could be linked to non-functional copies of *sdY* in the genome. We thereafter answered this by quantifying multiple *sdY* copies in the genome using qPCR on genomic DNA from 2,025 accurately phenotyped Atlantic salmon originating from 64 families of domesticated, F1-hybrid, and wild origin. We therefore demonstrate that the *sdY* gene has an infrequent non-functional copy in the genome, consistent with autosomal inheritance, which explains the observed discordance in Atlantic salmon females.

## Materials and Methods

### Experimental Crosses

Over the past decade, we have conducted a number of pedigree-controlled studies on a multiple-generation experimental population of domesticated and wild Atlantic salmon and their crosses at the aquaculture facility owned by the Institute of Marine Research located in Matre, western Norway (Solberg et al., 2013, 2014; Ayllon et al., 2015; Harvey et al., 2016, 2018; Glover et al., 2018, 2020; Perry et al., 2019; Besnier et al., 2020). The reader is directed to these publications for full details regarding the standard rearing conditions experienced in this fish farm. In the present study, we produced a total of 29 (F1-C2011) and 39 (F1-C2012) experimental families in the years 2011 and 2012, respectively. These families originated from the domesticated Mowi strain (13 families), the wild Figgjo population (14 families), reciprocal F1-hybrids between Mowi and Figgjo (24 families), the wild Vosso population (7 families), and the wild Arna population (6 families). Extensive details of these experimental crosses and the background of the source populations are available elsewhere (Solberg et al., 2014).

After fertilization in 2010 and 2011, eggs were incubated in single-family containers until the eyed stage when they were mixed into common-garden experiments to study a range of phenotypic traits (data not used here). These fish were first reared until smoltification in freshwater aged 1+ when 2,000 (F1-C2011) and 2,400 (F1-2012) individuals were PIT tagged and DNA sampled, and thereafter transferred into sea-cages where they were reared until they matured after a further 1–3 years. Families represented by less than 10 individuals at maturity were discarded. Upon maturation, the phenotypic sex of 2,025 individuals from 64 families was accurately recorded by dissection, giving a total of 1,048 and 977 phenotypically validated males and females, respectively.

### Genetic Analysis—Microsatellites and SNPs

Total DNA from all offspring and parents was purified using the Qiagen DNeasy Blood & Tissue Kit (Qiagen, Hilden, Germany) according to the manufacturer’s recommendations. Microsatellite DNA parentage testing was used to identify the pedigree of all individuals used in this study using the exclusion based method implemented in FAP (Taggart, 2007) using six microsatellites. Following the above-mentioned procedure, 97–99% of the offspring were unambiguously assigned to their family of origin. The laboratory conducting these analyses has extensive experience in DNA parentage testing (Solberg et al., 2013, 2014; Harvey et al., 2016, 2018; Glover et al., 2018), and the full details regarding the markers used and their amplification conditions are available in these previous studies.

In addition to microsatellites, a set of 116 genome-wide distributed SNPs were genotyped in all offspring and parents for the purpose of linkage mapping (see below). This analysis was performed on a MassARRAY Analyzer 4 from Agena Bioscience^TM^ according to the manufacturer’s instructions. The final dataset for mapping included 109 genome-wide distributed SNPs once those displaying poor coverage and clustering were removed. The list of SNPs are available elsewhere (Besnier et al., 2015, 2020) and their genomic location can be retrieved from the article describing the Atlantic salmon linkage map (Lien et al., 2011).

### PCR-Based *sdY* Tests

The *sdY* presence/absence was validated by a PCR-based methodology aimed to detect the presence of the *sdY* gene (Yano et al., 2012; Eisbrenner et al., 2014). Individuals showing amplicons of exon 2 and 4 were designated as males. As a positive PCR control and for species determination we used the presence of the 5S rRNA gene (Pendas et al., 1995). PCR amplifications were performed using reaction mixtures containing approximately 50 ng of extracted Atlantic salmon DNA, 10 nM Tris–HCl pH 8.8, 1.5 mM MgCl2, 50 mM KCl, 0.1% Triton X-100, 0.35 μM of each primers, 0.5 Units of DNA Taq Polymerase (Promega, Madison, WI, United States) and 250 μM of each dNTP in a final volume of 20 μL. PCR products were visualized in 3% agarose gels.

A quantitative PCR (qPCR) based methodology was developed to quantify the number of *sdY*-liked copies present. *gapdh*, *sdY* exon 2, and *sdY* exon4 were multiplexed using 5’labeled probes (Supplementary Table 1). The *gapdh* locus was used as an internal positive control (IPC) and reference gene to estimate fold change (FC) values (Livak and Schmittgen, 2001). Amplification reactions were run on a QuantStudio5 384 real time detection system (Thermo Fisher Scientific, United States). Reactions consisted of a Pre-Read stage (60°C for 30 s), a Hold Stage (95°C for 10 min), a PCR stage (40 cycles of 95°C for 15 s and 60°C for 1 min) and Post-Read stage (60°C for 30 s). Each 5 μl reaction contained the following final concentrations: 1× Taqman Universal MasterMix, 1 μM *gapdh* forward and reverse primers, 0.2 μM *gapdh* TaqMan probe, 1.4 μM *sdY*_Exon2 forward and reverse primers, 0.32 μM *sdY* _Exon2 TaqMan probe, 2.1 μM *sdY*_Exon4 forward and reverse primers, 0.48 μM *sdY*_Exon4 TaqMan probe and 2 ng/μl of gDNA template. Whenever possible, no template controls (NTC) and reference males and females were included.

In order to validate the qPCR methodology, we used XY males and YY super-males. XY males will carry a single copy of the Y-specific sex determining gene *sdY*. On the other hand, super males will carry two *sdY* gene copies, one per Y chromosome. YY males are the product of either self-fertilization or double haploid males. Full details on YY super-males production can be found in Fjelldal et al. (under review). Briefly, eggs and milt from a hermaphrodite salmon were surgically removed to prevent undesired self-fertilization. Eggs were then self-fertilized either with normal or UV-irradiated milt. Following the fertilization with UV-irradiated milt, pressure mediated diploidization was carried out to produce the double haploid males used in this study.

### Linkage Mapping

Linkage mapping was performed on all 64 families, including the eight families showing discrepancy between genetic and phenotypic sex. For each family, the coefficient of Identity By Descent (IBD) among offspring alleles was estimated from both pedigree and genotype information as in Pong-Wong et al. (2001). First, the genomic location of the sex determining locus was considered. The link between the binary phenotype (male/female) and the two paternally (maternally) inherited alleles was investigated by fitting a Chi-squared test in each family separately, at each SNP locus. Second, the genomic location of the sex discrepancy for the eight affected families was investigated following the same Chi-squared approach, also at the family level. Here, the two phenotypes were no longer male and female but discrepant/non-discrepant individuals, where the non-discrepant group consisted of all the regular males and females, and the discrepant group consisted of phenotypic females that amplified one or more copy of *sdY*, as well as phenotypic males that amplified more than one *sdY* copy.

### Statistical Analysis

Chi square tests with computed *p*-values by Monte Carlo simulations (10^6^ replicates) were used to test for deviations of the observed values from the expected *sdY* genotypes frequencies. All statistical analyses were conducted in R V.3.6.2. (R Development Core Team, 2019).

### Ethical Considerations and Research Permits

The experimental protocols (permit numbers 4268, 5296) were approved by the Norwegian Animal Research Authority (NARA). Use of experimental animals were performed in strict accordance with the Norwegian Animal Welfare Act. This included anesthesia or euthanasia of fish using metacain (Finquel^®^ Vet, ScanVacc, Årnes, Norway), during all described procedures. In addition, all personnel involved in this experiment had undergone training approved by the Norwegian Food Safety Authority, which is mandatory for all personnel running experiments involving animals included in the Animal Welfare Act.

## Results

**FIGURE 1:**
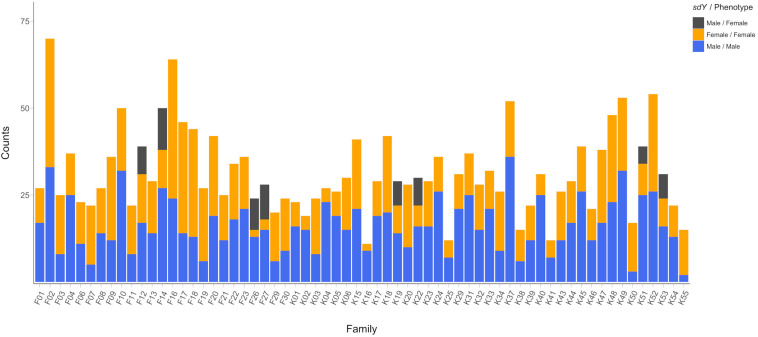
Phenotypic sex frequencies distribution across the 64 studied families showing PCR-based *sdY* genetic sex concordances. Concordant males and females are displayed in blue and orange, respectively. Discordant females (*sdY* positive phenotypic females) are shown in dark gray.

We found PCR-based discordance between the validated phenotypic sex and *sdY* genotype in 66 individuals out of the 2,025 fish from the 64 families tested (Figure 1). All of the reported cases were phenotypic females displaying a positive signal for *sdY*. Discordance between the *sdY* presence/absence and phenotypic sex was only observed in females from eight of the 64 families, ranging from 36 to 82% discordance among females per family. Of the 88 parents used as broodstock, three phenotypic females were *sdY* positive. These females were the mothers of families F26, F27 and K22, all of which had female offspring displaying discordance between phenotypic and genetic sex (Figure 1). The five other families containing discordant offspring did not have discordant mothers.

**FIGURE 2:**
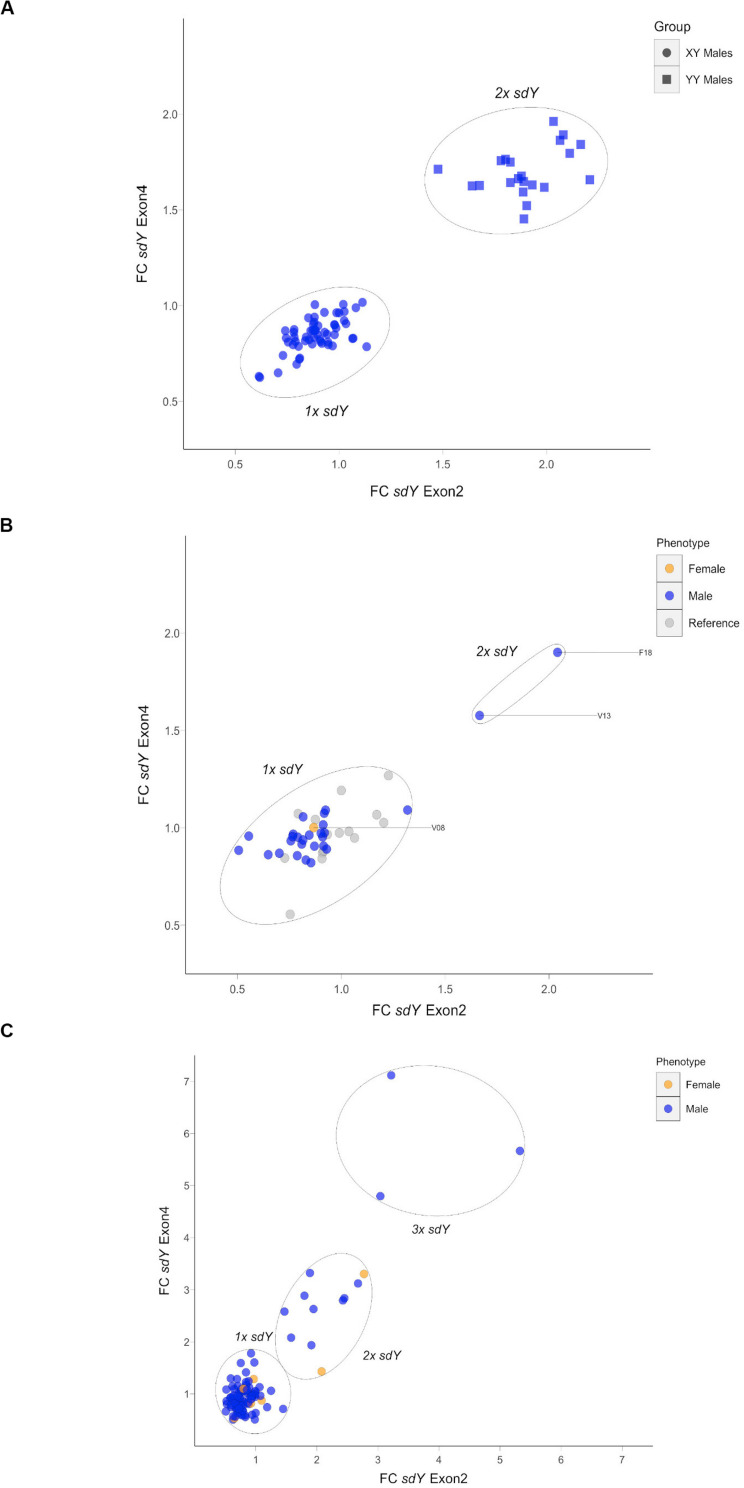
Real time PCR based fold change (FC) values for *sdY* exon2 and exon4 amplicons. **(A)** Validation RT PCR using XY males and YY super-males; round and square dots, respectively. **(B)** Parental individuals for the F1-K2012 families showing 1–2× *sdY* sires individuals, in blue, and the 1× *sdY* discordant dam in orange. Reference 1× *sdY* males are displayed in gray. **(C)** Example plate showing 1–3× *sdY* males and 1–2× *sdY* females in blue and orange, respectively, among the offspring. Reference males not displayed.

**FIGURE 3:**
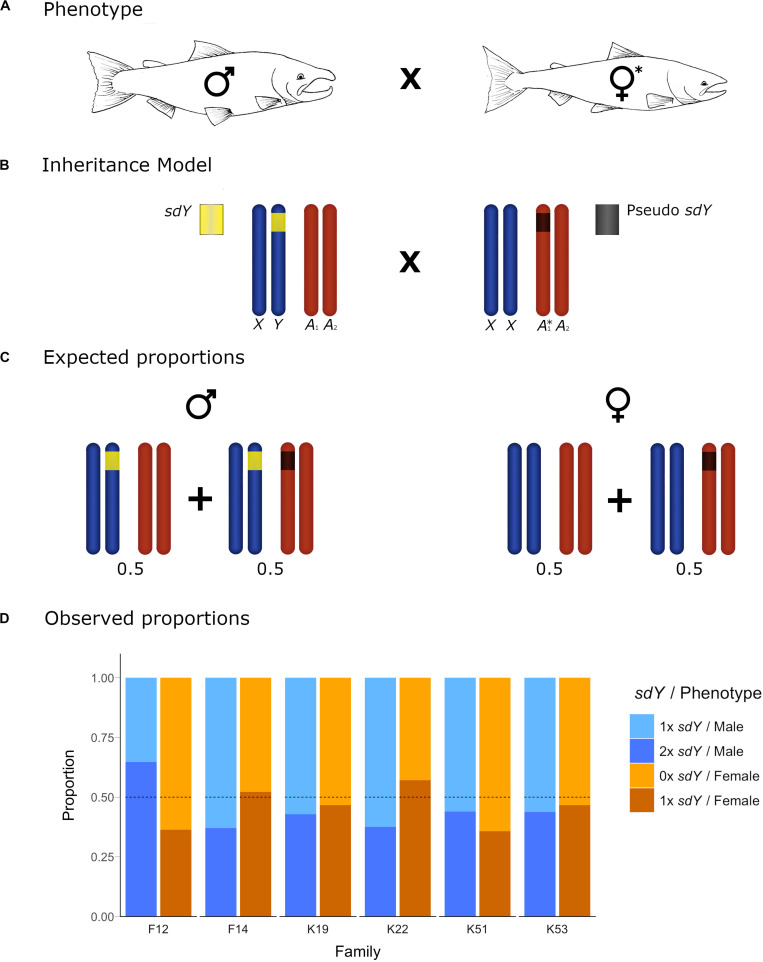
Conceptual diagram showing the inheritance model and the observed frequencies for the offspring of crosses with one parental individual carrying an *sdY* pseudocopy. **(A)** Cross between a 1× *sdY* phenotypic male and a 1× *sdY* discordant female denoted with ^∗^. **(B)** conceptual diagram showing the inheritance model at a chromosome level. Sex chromosomes and autosomes are represented in dark blue and dark orange, respectively. Normal 1× *sdY* male (left) carrying a copy of the sex determining *sdY* gene (yellow) in the Y chromosome. Discrepant 1× *sdY* phenotypic female (right) carrying an *sdY* autosomic pseudocopy (dark gray) in heterozygosis. **(C)** Males and females expected proportions for the different *sdY* genotypes (1–2×). **(D)** Offspring observed proportions for the affected families of 1× and 2× *sdY* males and 0× and 1× *sdY* females: light blue, blue, light orange and orange, respectively. Dotted lines represent the *sdY* genotype expected frequencies.

**FIGURE 4:**
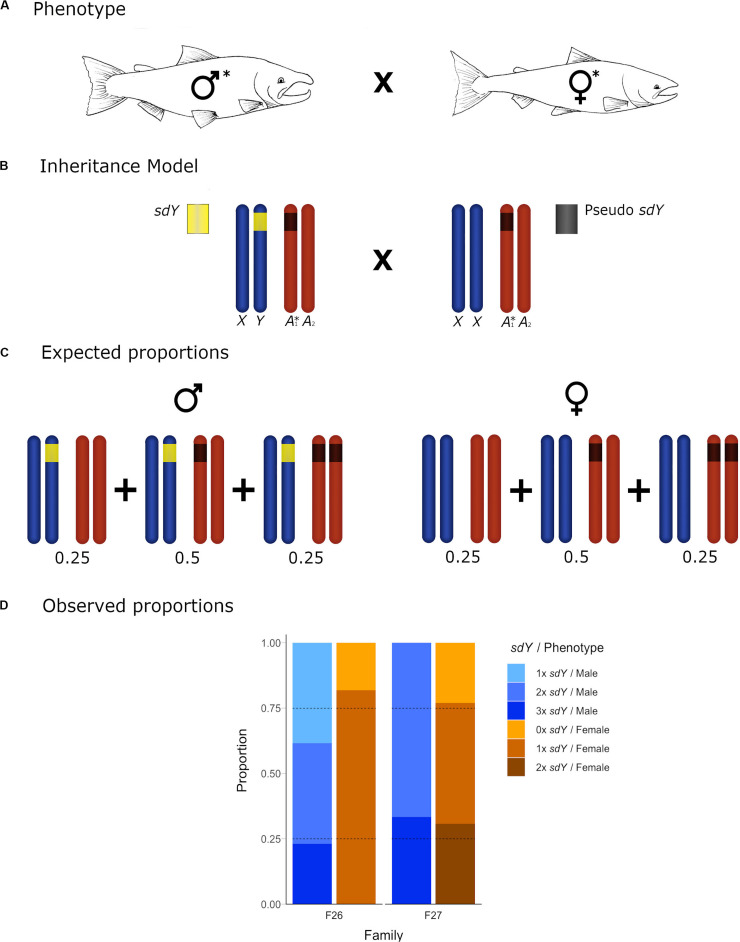
Conceptual diagram showing the inheritance model and the observed frequencies for the offspring of crosses with both parental individuals carrying an *sdY* pseudocopy. **(A)** Cross between a 2× *sdY* phenotypic male and a 1× *sdY* discordant female: Both affected parental individuals are denoted with an asterisk. **(B)** Conceptual diagram showing the inheritance model at a chromosome level. Sex chromosomes and autosomes are represented in dark blue and dark orange, respectively. **(B)** 2× *sdY* male (left) carrying a copy of the sex determining *sdY* gene (yellow) in the Y chromosome and the extra autosomic copy (dark gray) in heterozygosis. Discrepant 1× *sdY* phenotypic female (right) carrying an *sdY* autosomic pseudocopy (dark gray) in heterozygosis. **(C)** Males and females expected proportions for the different *sdY* genotypes (1–3×). **(D)** Offspring observed proportions for the affected families of 1–3× *sdY* males and 0–2× *sdY* females: light blue, blue, dark blue, light orange, orange, and dark orange, respectively. Dotted lines represent the *sdY* genotype expected frequencies.

Based on the above result, we hypothesized that some Atlantic salmon may display a second autosomic copy of *sdY* in the genome. To examine this possibility, we used FC values from the qPCR assay in order to investigate the number of copies of the *sdY* present in each individual (both the sex determining gene and the potential autosomic pseudocopy). First, we genotyped known XY and YY males in order to validate the potential to identify two copies of this gene using the assay (Figure 2A). This test demonstrated that XY males clustered around 1 FC values for both amplicons (exons 2 and 4). In addition, YY males, i.e., males containing two *sdY* copies, all clustered around a FC value of 2 (>1.5 FC threshold for both exons). All three discordant dams described above carried a single *sdY* copy while there were four sires that were identified as carrying two copies of *sdY* (Figure 2B). Significantly, these four males sired the five families displaying discordant offspring that did not have a discordant dam, and in addition, sired two families with a discordant dam (Supplementary Table 2). Thus, at this stage, it was demonstrated that all eight families displaying discordant offspring had one or two discordant parents. Furthermore, none of the 56 families without discordant offspring had discordant parents. Among the 2,025 offspring, 62 and 4 of the phenotypic females displayed one and two copies of *sdY*, respectively, and, 66 and eight of the phenotypic males displayed two and three copies of *sdY*, respectively (Figure 2C). All of these individuals were reported from the eight families with offspring displaying discordance between phenotypic and genetic sex. When these data were considered on a family by family basis, the observed frequencies of the offspring carrying a variable number of copies of the *sdY* gene were highly consistent with autosomal inheritance (Figures 3, 4).

Based upon our hypothesis developed above, and the observed parental *sdY* genotypes in six of the families, we expected to see a 50/50 frequency in the female offspring displaying 0× vs. 1× *sdY* copies, and the same frequency in male offspring displaying 1× vs. 2× *sdY* copies. The observed frequencies (Figure 3) did not deviate from the expected frequencies (Chi Square *p*-values ranging from 0.24 to 0.98). In the remaining two families, and based upon the parental genotypes, we expected to see a 25/50/25 distribution in the frequencies 0×, 1×, 2×, and 1×, 2×, or 3× copies of *sdY* for female and male offspring, respectively. Although the observed offspring frequencies did not match exactly with these expected frequencies (Figure 4), most likely due to very low N offspring within these families, they did not significantly deviate from the expected frequencies (*p*-values 0.29 and 0.34 from families 26 and 27, respectively).

Within the 64 families, validated phenotypic sex was mapped to chromosomes Ssa02, Ssa03 and Ssa06 (Supplementary Table 2). Thereafter, the offspring from the eight affected families, with their *sdY* genotype (i.e., females displaying 0× vs. 1× or 2× *sdY* copies, and males displaying 1× vs. 2× or 3× *sdY* copies), was mapped to chromosomes Ssa03, Ssa05, Ssa06, Ssa13 and Ssa28. Statistical support was however variable for some of the mapping data, in part possibly due to low N observations (Supplementary Table 2).

## Discussion

The discovery of the master sex-determining *sdY* gene and its function in salmonids represents a significant advance in knowledge (Yano et al., 2012, 2013; Bertho et al., 2018), opening up new avenues of research. However, phenotype*-sdY* discordances have been reported in many of the salmonid species, which has left open questions regarding *sdY* in salmonids (Yano et al., 2012; Cavileer et al., 2015; Larson et al., 2016). Here, we have presented extensive and compelling data that strongly suggest that these discordances in Atlantic salmon are caused by low-frequency *sdY* copies in the genome that are not involved in sex determination.

Within the genetic material studied here, we observed 6.75% discordant females, and a total of 4% of the individuals with a second or third copy of the pseudo *sdY* gene. These fish originated from three dams and five sires among the 88 parents. The number of discordant females observed here is higher than the 1% frequency observed in a domesticated Tasmanian Atlantic salmon strain (Eisbrenner et al., 2014; Kijas et al., 2018) but similar to the 7% recently reported for the same Tasmanian strain (Brown et al., 2020). Given the inheritance model presented here, it is likely that this difference is merely the result of the number of affected parents and the cross design, although strain specific differences in the frequency of the pseudocopy of *sdY* cannot be ruled out. The 7 and 12% discordances between phenotype and *sdY* marker for sex reported in sockeye salmon (Larson et al., 2016) and the chinook salmon (Cavileer et al., 2015), respectively, raise the question of whether the same phenomenon described in Atlantic salmon here is also the cause of observed discordance in the other salmonid species. Members of the Coregoninae subfamily lack *sdY* male specificity and the existence of sex specific inactive copies in females has been invoked to explain this phenomenon (Yano et al., 2012). Together with the mobile nature of the *sdY* gene, the loss of function may also explain the existence of inactive *sdY* copies in other salmonid species (Podlesnykh et al., 2017).

Since the *sdY* gene was discovered (Yano et al., 2013), different mechanisms have been invoked to explain the existence of discordant phenotypes such as phenotyping or sampling errors, environment mediated sex reversal, female-specific gene inactivation, sequence variability, the existence of minor sex determining (SD) genes and recombination (Yano et al., 2013; Cavileer et al., 2015; Larson et al., 2016; Eysturskarð et al., 2017; King and Stevens, 2020). Recently, a dosage-dependent mechanism has been suggested to explain these discrepancies in Atlantic salmon (Brown et al., 2020), suggesting that *sdY* is present in a single copy in the male genome and might be also present as partial copies in the female genome. However, our results strongly point to the existence of non-functional autosomal copies as previously suggested for two coregoninae species (Yano et al., 2013) and sockeye salmon (Larson et al., 2016). Here we report the existence of phenotypic females with up to two full *sdY* autosomal copies which appear to have lost their ability to function as a proper SD gene causing the apparent discordance.

Within Atlantic salmon, *sdY* has been mapped to chromosomes Ssa02, Ssa03 and Ssa06 in domesticated and wild strain of salmon from North America and Norway (Eisbrenner et al., 2014; Kijas et al., 2018; Besnier et al., 2020). It has also been mapped to chromosomes Ssa02 and Ssa21 in six wild Spanish populations (Gabian et al., 2019). The above observations are consistent with the findings of the present study where the SD *sdY* was mapped to Ssa02, Ssa03, and Ssa06 in the 64 families. In all of these studies, chromosome Ssa02 was identified as the most common location for *sdY* and is likely to be the ancestral variant (Kijas et al., 2018). Surprisingly, low divergence between the Ssa03 and Ssa06 loci has been reported (Kijas et al., 2018), suggesting a recent origin even though these variants are present in both the North American and European lineages. Here, we mapped *sdY* autosomal copies to chromosomes Ssa03, Ssa05, Ssa06, Ssa13, and Ssa28. Interestingly however, within the eight families displaying *sdY* autosomal copies, SD *sdY*, and autosomal *sdY* were only mapped on the same chromosome in family F12. Therefore, these two genes typically did not co-locate on chromosomes in the analyzed families. Ultimately, this might explain why only a limited number of chromosomes are recursively recruited as sex chromosomes in the species (Eisbrenner et al., 2014; Gabian et al., 2019), both recently and in different lineages (Kijas et al., 2018). The primers used here have been proven to be robust in detecting both *sdY* and its autosomal copy at the expected frequencies, so it is reasonable to infer a high degree of primer binding sequence conservation. Hence, it is also fair to reason that these pseudocopies may very well be the product of recent transpositions (Lubieniecki et al., 2015) and may constitute failed attempts to recruit novel sex determining loci in the species. Sex chromosome recruitment in Atlantic salmon may be the product of the transposable nature of the *sdY* gene (Faber-Hammond et al., 2015) and gene landscape (Bertho et al., 2018), which might explain the loss of function of the autosomal copies reported here. A functional *sdY* copy is considered necessary for maleness in salmonids but Atlantic salmon *sdY* negative males has been sporadically reported (Perry et al., 2019; Brown et al., 2020). However, they seem to be the product of PCR artifacts (King and Stevens, 2020).

Results of the present study, including the *sdY* copy number assay developed herein, have implications for commercial salmonid breeding programs. Breeders are increasingly using the *sdY* gene to determine phenotypic sex and to assist broodstock selection in the early production phase. Discordance between phenotypic and *sdY* based genetic tests has been reported for example in the commercial Mowi strain representing a logistic and financial challenge (Matt Baranski, Mowi, personal communication). Being able to identify both males and females carrying pseudocopies creates the opportunity to remove this pseudocopy from the breeding line in one generation: males carrying two or three copies and females carrying two copies can be removed early, and single copy carrying females weeded out when the phenotype is clear. Additionally, gaining knowledge about the proper genomic environment needed for a successful sex chromosome recruitment might constitute a huge leap in the race of understanding the precise mechanisms behind sex determination and ultimately in gaining control of the process from an aquaculture perspective.

## Data Availability Statement

The data is available at Open Science Framework with the next Identifier: DOI: 10.17605/OSF.IO/3XHA9 (https://osf.io/3xha9/).

## Ethics Statement

The animal study was reviewed and approved by the Norwegian Animal Research Authority (NARA) Permit numbers 4268 and 5296.

## Author Contributions

KG, MS, and FA conceived and designed the experiments. FA, MS, and PF performed the experiments. FA, MS, FB, and KG analyzed the data and contributed to the interpretation of the results. KG, AW, RE, PF, and TH contributed to the reagents, materials, and analysis tools. FA and KG wrote the manuscript. All authors provided critical feedback and helped shape the research, analysis, and manuscript.

## Conflict of Interest

The authors declare that the research was conducted in the absence of any commercial or financial relationships that could be construed as a potential conflict of interest.

## References

[B1] Ashman T.-L. Bachtrog D. Blackmon H. Goldberg E. E. Hahn M. W. Kirkpatrick M. (2014). Tree of sex: a database of sexual systems. *Sci. Data* 1:140015. 10.1038/sdata.2014.15 25977773 PMC4322564

[B2] Ayllon F. Kjaerner-Semb E. Furmanek T. Wennevik V. Solberg M. F. Dahle G. (2015). The *vgll3* locus controls age at maturity in wild and domesticated Atlantic salmon (*Salmo salar* L.) males. *PLoS Genet.* 11:e1005628. 10.1371/journal.pgen.1005628 26551894 PMC4638356

[B3] Ayllon F. Solberg M. F. Besnier F. Fjelldal P. G. Hansen T. J. Wargelius A. (2020). Sex determining gene transposition as an evolutionary platform for chromosome turnover. *bioRxiv* 10.1101/2020.03.14.991026

[B4] Ayllon F. Solberg M. F. Glover K. A. Mohammadi F. Kjaerner-Semb E. Fjelldal P. G. (2019). The influence of *vgll3* genotypes on sea age at maturity is altered in farmed *mowi* strain Atlantic salmon. *BMC Genet.* 20:44. 10.1186/s12863-019-0745-9 31060499 PMC6501413

[B5] Barson N. J. Aykanat T. Hindar K. Baranski M. Bolstad G. H. Fiske P. (2015). Sex-dependent dominance at a single locus maintains variation in age at maturity in salmon. *Nature* 528 405–408. 10.1038/nature16062 26536110

[B6] Bertho S. Herpin A. Branthonne A. Jouanno E. Yano A. Nicol B. (2018). The unusual rainbow trout sex determination gene hijacked the canonical vertebrate gonadal differentiation pathway. *Proc. Natl. Acad. Sci. U.S.A.* 115 12781–12786. 10.1073/pnas.1803826115 30463951 PMC6294932

[B7] Besnier F. Glover K. A. Lien S. Kent M. Hansen M. M. Shen X. (2015). Identification of quantitative genetic components of fitness variation in farmed, hybrid and native salmon in the wild. *Heredity* 115 47–55. 10.1038/hdy.2015.15 26059968 PMC4815496

[B8] Besnier F. Solberg M. F. Harvey A. C. Carvalho G. R. Bekkevold D. Taylor M. I. (2020). Epistatic regulation of growth in Atlantic salmon revealed: a QTL study performed on the domesticated-wild interface. *BMC Genet.* 21:13. 10.1186/s12863-020-0816-y 32033538 PMC7006396

[B9] Brown M. S. Evans B. S. Afonso L. O. B. (2020). Discordance for genotypic sex in phenotypic female Atlantic salmon (*Salmo salar*) is related to a reduced *sdY* copy number. *Sci. Rep.* 10:9651 10.1038/s41598-020-66406-x PMC7296011 32541863

[B10] Cavileer T.D. Hunter S.S. Olsen J. Wenburg J. Nagler J.J. (2015). A sex-determining gene (*sdY*) assay shows discordance between phenotypic and genotypic sex in wild populations of chinook salmon. *Trans. Am. Fish. Soc.* 144 423–430. 10.1080/00028487.2014.993479

[B11] Cnaani A. Lee B. Y. Zilberman N. Ozouf-Costaz C. Hulata G. Ron M. (2008). Genetics of sex determination in tilapiine species. *Sex. Dev.* 2 43–54. 10.1159/000117718 18418034

[B12] Eisbrenner W. S. Botwright N. Cook M. Davidson E. A. Dominik S. Elliott N. G. (2014). Evidence for multiple sex-determining loci in Tasmanian Atlantic salmon (*Salmo salar*). *Heredity* 113 86–92. 10.1038/hdy.2013.55 23759729 PMC4815647

[B13] Eysturskarð J. Dam M. í Kongsstovu S. K. Jacobsen Á Petersen P. E. (2017). Rapid sex identification of Atlantic salmon (*Salmo salar* L.) by real-time PCR. *Aquac. Res.* 48 2618–2620. 10.1111/are.13003

[B14] Faber-Hammond J. J. Phillips R. B. Brown K. H. (2015). Comparative analysis of the shared sex-determination region (SDR) among salmonid fishes. *Genome Biol. Evol.* 7 1972–1987. 10.1093/gbe/evv123 26112966 PMC4524489

[B15] Gabian M. Moran P. Fernandez A. I. Villanueva B. Chtioui A. Kent M. P. (2019). Identification of genomic regions regulating sex determination in Atlantic salmon using high density SNP data. *BMC Genomics* 20:764. 10.1186/s12864-019-6104-4 31640542 PMC6805462

[B16] Glover K. A. Harvey A. C. Hansen T. J. Fjelldal P. G. Besnier F. N. Bos J. B. (2020). Chromosome aberrations in pressure-induced triploid Atlantic salmon. *BMC Genet.* 21:59. 10.1186/s12863-020-00864-0 32505176 PMC7276064

[B17] Glover K. A. Solberg M. F. Besnier F. Skaala O. (2018). Cryptic introgression: evidence that selection and plasticity mask the full phenotypic potential of domesticated Atlantic salmon in the wild. *Sci. Rep.* 8:13966. 10.1038/s41598-018-32467-2 30228303 PMC6143624

[B18] Guyomard R. Guiguen Y. Bernard M. Charlet A. Dechamp N. Hervet C. (2014). “RAD-seq mapping of spontaneous masculinization in XX doubled haploid rainbow trout lines,” in *Proceedings of the 10th World Congress on Genetics Applied to Livestock Production (WCGALP)*), Vancouver, BC.

[B19] Harvey A. C. Skilbrei O. T. Besnier F. Solberg M. F. Sorvik A. G. E. Glover K. A. (2018). Implications for introgression: has selection for fast growth altered the size threshold for precocious male maturation in domesticated Atlantic salmon? *BMC Evol. Biol.* 18:188. 10.1186/s12862-018-1294-y 30558529 PMC6298023

[B20] Harvey A. C. Solberg M. F. Troianou E. Carvalho G. R. Taylor M. I. Creer S. (2016). Plasticity in growth of farmed and wild Atlantic salmon: is the increased growth rate of farmed salmon caused by evolutionary adaptations to the commercial diet? *BMC Evol. Biol.* 16:264. 10.1186/s12862-016-0841-7 27905882 PMC5134087

[B21] Hattori R. S. Somoza G. M. Fernandino J. I. Colautti D. C. Miyoshi K. Gong Z. (2019). The duplicated Y-specific *amhy* gene is conserved and linked to maleness in silversides of the genus *Odontesthes*. *Genes* 10:679. 10.3390/genes10090679 31491991 PMC6770987

[B22] Heule C. Salzburger W. Bohne A. (2014). Genetics of sexual development: an evolutionary playground for fish. *Genetics* 196 579–591. 10.1534/genetics.114.161158 24653206 PMC3948791

[B23] Kijas J. McWilliam S. Sanchez M. N. Kube P. King H. Evans B. (2018). Evolution of sex determination loci in Atlantic salmon. *Sci. Rep.* 8:5664. 10.1038/s41598-018-23984-1 29618750 PMC5884791

[B24] King R. A. Stevens J. R. (2020). An improved genetic sex test for Atlantic salmon (*Salmo salar* L.). *Conserv. Genet. Resour.* 12 191–193. 10.1007/s12686-019-01094-y

[B25] Kjaerner-Semb E. Ayllon F. Furmanek T. Wennevik V. Dahle G. Niemela E. (2016). Atlantic salmon populations reveal adaptive divergence of immune related genes - a duplicated genome under selection. *BMC Genomics* 17:610. 10.1186/s12864-016-2867-z 27515098 PMC4982270

[B26] Kusche H. Côté G. Hernandez C. Normandeau E. Boivin-Delisle D. Bernatchez L. (2017). Characterization of natural variation in North American Atlantic salmon populations (Salmonidae: *Salmo salar*) at a locus with a major effect on sea age. *Ecol. Evol.* 7 5797–5807. 10.1002/ece3.3132 28808549 PMC5550958

[B27] Larson W. A. McKinney G. J. Seeb J. E. Seeb L. W. (2016). Identification and characterization of sex-associated loci in sockeye salmon using genotyping-by-sequencing and comparison with a sex-determining assay based on the *sdY* gene. *J. Hered.* 107 559–566. 10.1093/jhered/esw043 27417855

[B28] Lien S. Gidskehaug L. Moen T. Hayes B. J. Berg P. R. Davidson W. S. (2011). A dense SNP-based linkage map for Atlantic salmon (*Salmo salar*) reveals extended chromosome homeologies and striking differences in sex-specific recombination patterns. *BMC Genomics* 12:615. 10.1186/1471-2164-12-615 22182215 PMC3261913

[B29] Lien S. Koop B. F. Sandve S. R. Miller J. R. Kent M. P. Nome T. (2016). The Atlantic salmon genome provides insights into rediploidization. *Nature* 533 200–205. 10.1038/nature17164 27088604 PMC8127823

[B30] Livak K. J. Schmittgen T. D. (2001). Analysis of relative gene expression data using real-time quantitative PCR and the 2(T)(-Delta Delta C) method. *Methods* 25 402–408. 10.1006/meth.2001.1262 11846609

[B31] Lubieniecki K. P. Lin S. Cabana E. I. Li J. Y. Lai Y. Y. Y. Davidson W. S. (2015). Genomic instability of the sex-determining locus in Atlantic salmon (*Salmo salar*). *G3 (Bethesda)* 5 2513–2522. 10.1534/g3.115.020115 26401030 PMC4632069

[B32] Macqueen D. J. Johnston I. A. (2014). A well-constrained estimate for the timing of the salmonid whole genome duplication reveals major decoupling from species diversification. *Proc. R. Soc. B-Biol. Sci.* 281:20132881. 10.1098/rspb.2013.2881 24452024 PMC3906940

[B33] Metcalf V. J. Gemmell N. J. (2006). Sexual genotype markers absent from small numbers of male New Zealand Oncorhynchus tshawytscha. *J. Fish Biol.* 68 136–143. 10.1111/j.0022-1112.2006.00903.x

[B34] Nagler J. J. Bouma J. Thorgaard G. H. Dauble D. D. (2001). High incidence of a male-specific genetic marker in phenotypic female chinook salmon from the Columbia River. *Environ. Health Perspect.* 109 67–69. 10.1289/ehp.0110967 11171527 PMC1242053

[B35] Pendas A. M. Moran P. Martinez J. L. Garciavazquez E. (1995). Applications of 5S-rDNA in Atlantic salmon, brown trout, and in Atlantic salmon x brown trout hybrid identification. *Mol. Ecol.* 4 275–276. 10.1111/j.1365-294X.1995.tb00220.x 7735532

[B36] Pennell M. W. Mank J. E. Peichel C. L. (2018). Transitions in sex determination and sex chromosomes across vertebrate species. *Mol. Ecol.* 27 3950–3963. 10.1111/mec.14540 29451715 PMC6095824

[B37] Perry W. B. Solberg M. F. Besnier F. Dyrhovden L. Matre I. H. Fjelldal P. G. (2019). Evolutionary drivers of kype size in Atlantic salmon (*Salmo salar*): domestication, age and genetics. *R. Soc. Open Sci.* 6:190021. 10.1098/rsos.190021 31183145 PMC6502380

[B38] Phillips R. B. (2013). Evolution of the sex chromosomes in salmonid fishes. *Cytogenet. Genome Res.* 141 177–185. 10.1159/000355149 24107355

[B39] Podlesnykh A. V. Brykov V. A. Kukhlevsky A. D. (2017). Unstable linkage of molecular markers with sex determination gene in Pacific salmon (*Oncorhynchus* spp.). *J. Hered.* 108 328–333. 10.1093/jhered/esx001 28391306

[B40] Pong-Wong R. George A. W. Woolliams J. A. Haley C. S. (2001). A simple and rapid method for calculating identity-by-descent matrices using multiple markers. *Genet. Sel. Evol.* 33 453–471. 10.1186/1297-9686-33-5-453 11712969 PMC2705399

[B41] Qian W. F. Zhang J. Z. (2014). Genomic evidence for adaptation by gene duplication. *Genome Res.* 24 1356–1362. 10.1101/gr.172098.114 24904045 PMC4120088

[B42] R Development Core Team (2019). *R: A Language and Environment for Statistical Computing.* Vienna: R Foundation for Statistical Computing.

[B43] Ross J. A. Urton J. R. Boland J. Shapiro M. D. Peichel C. L. (2009). Turnover of sex chromosomes in the stickleback fishes (Gasterosteidae). *PLoS Genet.* 5:e1000391. 10.1371/journal.pgen.1000391 19229325 PMC2638011

[B44] Solberg M. F. Fjelldal P. G. Nilsen F. Glover K. A. (2014). Hatching time and alevin growth prior to the onset of exogenous feeding in farmed, wild and hybrid Norwegian Atlantic salmon. *PLoS One* 9:e113697. 10.1371/journal.pone.0113697 25438050 PMC4249964

[B45] Solberg M. F. Glover K. A. Nilsen F. Skaala Ø (2013). Does domestication cause changes in growth reaction norms? A study of farmed, wild and hybrid Atlantic salmon families exposed to environmental stress. *PLoS One* 8:e54469. 10.1371/journal.pone.0054469 23382901 PMC3561353

[B46] Taggart J. B. (2007). FAP: an exclusion-based parental assignment program with enhanced predictive functions. *Mol. Ecol. Notes* 7 412–415. 10.1111/j.1471-8286.2006.01616.x

[B47] Williamson K. S. May B. (2002). Incidence of phenotypic female chinook salmon positive for the male Y-chromosome-specific marker *OtY1* in the Central Valley, California. *J. Aquat. Anim. Health* 14 176–183. 10.1577/1548-86672002014<0176:IOPFCS<2.0.CO;2

[B48] Yano A. Guyomard R. Nicol B. Jouanno E. Quillet E. Klopp C. (2012). An immune-related gene evolved into the master sex-determining gene in *rainbow trout*, *Oncorhynchus mykiss*. *Curr. Biol.* 22 1423–1428. 10.1016/j.cub.2012.05.045 22727696

[B49] Yano A. Nicol B. Jouanno E. Quillet E. Fostier A. Guyomard R. (2013). The sexually dimorphic on the Y-chromosome gene (*sdY*) is a conserved male-specific Y-chromosome sequence in many salmonids. *Evol. Appl.* 6 486–496. 10.1111/eva.12032 23745140 PMC3673476

