# Supplementary material for: Autosomal sdY Pseudogenes Explain Discordances Between Phenotypic Sex and DNA Marker for Sex Identification in Atlantic Salmon
Source: Front Genet. 2020 Oct 14;11:544207. doi: 10.3389/fgene.2020.544207 (PMC7591749; doi:10.3389/fgene.2020.544207)
Supplement: Supplementary file 2 [file Data_Sheet_2.docx]

**Supplemental Tables**

**Supp. Table 1.** Primers and custom 5´ labeled probes used for real time PCR genetic sex determination. 6-Fam, VIC and NED reported dyes were used for *gapdh*, sdY exon 2 and 4 respectively. *gapdh* was used as internal positive control (IPC) and reference for fold change calculations.

| **Name** |  | **Sequence** | **Type** | **Task** |
| --- | --- | --- | --- | --- |
| Ss_ *gapdh* _F |  | 5´- CCGCCACCCAGAAGACTGT - 3´ | Forward Primer | IPC |
| Ss_ *gapdh* _R |  | 5´- CTGGCGCCACGTCCAT - 3´ | Reverse Primer |  |
| Ss_ *gapdh* _P |  | 6-FAM -TCCTTCTGGAAAGCTGTGGA - MGBNFQ | MGB Probe |  |
|  |  |  |  |  |
| Ss_*sdY*_Exon2_F |  | 5´- AGCCCCCCGACACCAT - 3´ | Forward Primer | Target |
| Ss_ *sdY* _Exon2_R |  | 5´- TTCAATCCTCTCCAACCTGCTT - 3´ | Reverse Primer |  |
| Ss_ *sdY* _Exon2_P |  | VIC – CCCATCTCCTCTGACC - MGBNFQ | MGB Probe |  |
|  |  |  |  |  |
| Ss_ *sdY* _Exon4_F |  | 5´- CCATGGGCTCAGCAGCTATT - 3´ | Forward Primer | Target |
| Ss_ *sdY* _Exon4_R |  | 5´- AGGATCTGGCTTGAGTCCTCC - 3´ | Reverse Primer |  |
| Ss_ *sdY* _Exon4_P |  | NED - AAGCAAGCTCACGACTT - MGBNFQ | MGB Probe |  |

**Supp. Table 2.** Overview of the 64 families including: family unique identifier (ID), strain origin (Type), Dam and sire unique identifier (Dam ID and Sire ID respectively), total number of individuals per family (N) and Dam and sire *sdY* copy number (Dam *sdY* and Sire *sdY* respectively). Additionally, linkage analysis results for the actual sex determining *sdY* locus (SEX) and the pseudocopy with *P* values. NA values are provided when analysis could not be performed either due to low N or insufficient genetic variation.

| Family | | | | | | |  | | SEX Linkage Analysis | |  | *sdY* Pseudocopy Linkage Analysis | |
| --- | --- | --- | --- | --- | --- | --- | --- | --- | --- | --- | --- | --- | --- |
| ID | Type | Dam ID | Sire ID | N | *sdY* Dam | *sdY* Sire | |  | Chromosome | *P* value |  | Chromsome | *P* value |
| F01 | Farm | #01 | #11 | 27 | 0x | 1x |  | | Ssa02 | 2E-10 |  | NA | NA |
| F02 | Hybrid | #01 | #31 | 70 | 0x | 1x |  | | Ssa03 | 5E-08 |  | NA | NA |
| F03 | Farm | #02 | #12 | 25 | 0x | 1x |  | | NA | NA |  | NA | NA |
| F04 | Hybrid | #02 | #32 | 37 | 0x | 1x |  | | NA | NA |  | NA | NA |
| F06 | Hybrid | #03 | #32 | 23 | 0x | 1x |  | | NA | NA |  | NA | NA |
| F07 | Farm | #04 | #14 | 22 | 0x | 1x |  | | Ssa02 | 2E-20 |  | NA | NA |
| F08 | Hybrid | #04 | #33 | 27 | 0x | 1x |  | | Ssa06 | 4E-10 |  | NA | NA |
| F09 | Farm | #05 | #15 | 36 | 0x | 1x |  | | Ssa02 | 2E-15 |  | NA | NA |
| F10 | Hybrid | #05 | #33 | 50 | 0x | 1x |  | | Ssa06 | 4E-15 |  | NA | NA |
| F11 | Farm | #06 | #16 | 22 | 0x | 1x |  | | Ssa02 | 3E-20 |  | NA | NA |
| F12 | Hybrid | #06 | #34 | 39 | 0x | 2x |  | | Ssa02/Ssa03 | 2E-06/3E-06 |  | Ssa03 | 6E-05 |
| F13 | Farm | #07 | #17 | 29 | 0x | 1x |  | | Ssa02 | 7E-09 |  | NA | NA |
| F14 | Hybrid | #07 | #35 | 50 | 0x | 2x |  | | Ssa02 | 8E-05 |  | Ssa06 | 3E-4 |
| F16 | Hybrid | #08 | #36 | 64 | 0x | 1x |  | | Ssa03 | 3E-16 |  | NA | NA |
| F17 | Farm | #09 | #19 | 46 | 0x | 1x |  | | Ssa02 | 2E-11 |  | NA | NA |
| F18 | Hybrid | #09 | #37 | 44 | 0x | 1x |  | | Ssa06 | 2E-05 |  | NA | NA |
| F19 | Farm | #10 | #20 | 27 | 0x | 1x |  | | Ssa02 | 7E-14 |  | NA | NA |
| F20 | Hybrid | #10 | #38 | 42 | 0x | 1x |  | | NA | NA |  | NA | NA |
| F21 | Wild | #21 | #31 | 25 | 0x | 1x |  | | Ssa03 | 1E-02 |  | NA | NA |
| F22 | Wild | #22 | #32 | 34 | 0x | 1x |  | | Ssa06 | 1E-02 |  | NA | NA |
| F23 | Wild | #23 | #33 | 36 | 0x | 1x |  | | Ssa06 | 4E-10 |  | NA | NA |
| F26 | Wild | #26 | #35 | 24 | 1x | 2x |  | | Ssa02 | 1E-02 |  | Ssa28 | 1E-03 |
| F27 | Wild | #27 | #35 | 28 | 1x | 2x |  | | Ssa02 | 2E-17 |  | Ssa06 | 3E-02 |
| F29 | Wild | #29 | #37 | 20 | 0x | 1x |  | | Ssa06 | 2E-04 |  | NA | NA |
| F30 | Wild | #30 | #38 | 24 | 0x | 1x |  | | Ssa06 | 2E-03 |  | NA | NA |
| K01 | Wild | A01 | A05 | 23 | 0x | 1x |  | | Ssa02 | 1E-02 |  | NA | NA |
| K02 | Wild | A01 | A06 | 19 | 0x | 1x |  | | Ssa06 | 6E-05 |  | NA | NA |
| K03 | Wild | A02 | A07 | 24 | 0x | 1x |  | | Ssa06 | 2E-16 |  | NA | NA |
| K04 | Wild | A02 | A08 | 27 | 0x | 1x |  | | Ssa03 | 1E-04 |  | NA | NA |
| K05 | Wild | A03 | A09 | 26 | 0x | 1x |  | | Ssa02 | 2E-14 |  | NA | NA |
| K06 | Wild | A04 | A10 | 30 | 0x | 1x |  | | Ssa06 | 2E-13 |  | NA | NA |
| K15 | Wild | V01 | V09 | 41 | 0x | 1x |  | | Ssa02 | 1E-13 |  | NA | NA |
| K16 | Wild | V02 | V10 | 11 | 0x | 1x |  | | Ssa02 | 3E-15 |  | NA | NA |
| K17 | Wild | V03 | V11 | 29 | 0x | 1x |  | | Ssa06 | 4E-06 |  | NA | NA |
| K18 | Wild | V04 | V12 | 42 | 0x | 1x |  | | NA | NA |  | NA | NA |
| K19 | Wild | V05 | V13 | 42 | 0x | 2x |  | | NA | NA |  | NA | NA |
| K20 | Wild | V06 | V14 | 28 | 0x | 1x |  | | Ssa02 | 2E-15 |  | NA | NA |
| K22 | Wild | V08 | V16 | 30 | 1x | 1x |  | | Ssa02 | 1E-02 |  | Ssa06 | 6E-03 |
| K23 | Wild | F01 | F10 | 29 | 0x | 1x |  | | Ssa06 | 7E-04 |  | NA | NA |
| K24 | Hybrid | F01 | M09 | 36 | 0x | 1x |  | | Ssa06 | 1E-08 |  | NA | NA |
| K25 | Hybrid | M01 | F10 | 12 | 0x | 1x |  | | Ssa06 | 2E-08 |  | NA | NA |
| K29 | Hybrid | M2 | F12 | 31 | 0x | 1x |  | | Ssa06 | 3E-07 |  | NA | NA |
| K31 | Wild | F03 | F13 | 37 | 0x | 1x |  | | Ssa02/Ssa06 | 2E-07/3E-06 |  | NA | NA |
| K32 | Hybrid | F03 | M11 | 28 | 0x | 1x |  | | Ssa02 | 2E-14 |  | NA | NA |
| K33 | Hybrid | M3 | F13 | 32 | 0x | 1x |  | | Ssa02 | 3E-30 |  | NA | NA |
| K34 | Farm | M03 | M11 | 26 | 0x | 1x |  | | Ssa02 | 5E-25 |  | NA | NA |
| K37 | Hybrid | M04 | F14 | 52 | 0x | 1x |  | | Ssa06 | 3E-19 |  | NA | NA |
| K38 | Farm | M04 | M12 | 15 | 0x | 1x |  | | Ssa02 | 2E-10 |  | NA | NA |
| K39 | Wild | F05 | F15 | 22 | 0x | 1x |  | | Ssa02 | 8E-06 |  | NA | NA |
| K40 | Hybrid | F05 | M13 | 31 | 0x | 1x |  | | Ssa02 | 7E-12 |  | NA | NA |
| K41 | Hybrid | M05 | F15 | 12 | 0x | 1x |  | | Ssa02 | 2E-08 |  | NA | NA |
| K43 | Wild | F06 | F16 | 26 | 0x | 1x |  | | NA | NA |  | NA | NA |
| K44 | Hybrid | F06 | M14 | 29 | 0x | 1x |  | | Ssa03 | 2E-04 |  | NA | NA |
| K45 | Hybrid | M6 | F16 | 39 | 0x | 1x |  | | NA | NA |  | NA | NA |
| K46 | Farm | M6 | M14 | 21 | 0x | 1x |  | | Ssa03 | 2E-09 |  | NA | NA |
| K47 | Wild | F07 | F17 | 38 | 0x | 1x |  | | Ssa02 | 4E-25 |  | NA | NA |
| K48 | Hybrid | F07 | M15 | 48 | 0x | 1x |  | | NA | NA |  | NA | NA |
| K49 | Hybrid | M7 | F17 | 53 | 0x | 1x |  | | Ssa02 | 2E-30 |  | NA | NA |
| K50 | Farm | M07 | M15 | 17 | 0x | 1x |  | | Ssa06 | 2E-02 |  | NA | NA |
| K51 | Wild | F08 | F18 | 39 | 0x | 2x |  | | Ssa02 | 2E-04 |  | Ssa05 | 1E-03 |
| K52 | Hybrid | F08 | M16 | 54 | 0x | 1x |  | | Ssa02 | 8E-13 |  | NA | NA |
| K53 | Hybrid | M08 | F18 | 31 | 0x | 2x |  | | Ssa02 | 3E-04 |  | Ssa13 | 2E-03 |
| K54 | Farm | M8 | M16 | 22 | 0x | 1x |  | | Ssa02 | 3E-04 |  | NA | NA |
| K55 | Wild | F09 | F11 | 15 | 0x | 1x |  | | NA | NA |  | NA | NA |
